# Adult Vaccination as a Protective Factor for Dementia: A Meta-Analysis and Systematic Review of Population-Based Observational Studies

**DOI:** 10.3389/fimmu.2022.872542

**Published:** 2022-05-03

**Authors:** Xinhui Wu, Haixia Yang, Sixian He, Ting Xia, Diang Chen, Yexin Zhou, Jin Liu, MengSi Liu, Zhen Sun

**Affiliations:** ^1^ Department of Geriatric, Hospital of Chengdu University of Traditional Chinese Medicine, Chengdu, China; ^2^ The General Hospital of Western Theater Command, Chengdu, China; ^3^ TCM Regulating Metabolic Diseases Key Laboratory of Sichuan Province, Hospital of Chengdu University of Traditional Chinese Medicine, Chengdu, China; ^4^ Clinical Medicine Teaching Department, Hospital of Chengdu University of Traditional Chinese Medicine, Chengdu, China; ^5^ The First Clinical Medical College, Guangxi University of Chinese Medicine, Guangxi, China; ^6^ Xi’an Hospital of Traditional Chinese Medicine, Xi’an, China; ^7^ Hengyang Medical School, University of South China, Hengyang, China

**Keywords:** vaccination, dementia, epidemiology, meta-analysis, systematic review, protective factor

## Abstract

**Background:**

Common vaccinations may have impacts on dementia risk, but current evidence is inconsistent. We therefore investigated the association between vaccinations and dementia risk by systematic review and meta-analysis approach.

**Methods:**

We conducted an extensive search of PubMed, Embase, Cochrane Library, and Web of Science to identify studies that compared the risk of dementia in vaccinated versus unvaccinated populations. The adjusted hazard ratio (HR) and corresponding 95% confidence intervals (CIs) were pooled as measures.

**Results:**

Of the 9124 records initially retrieved, 17 studies with 1857134 participants were included in our analysis. The overall pooled results showed that vaccinations were associated with a 35% lower dementia risk (HR=0.65, 95% CI: 0.60-0.71, *P*_overall effect_ < 0.001; *I^2 =^* 91.8%, *P*_heterogeneity_<0.001). All types of vaccination were associated with a trend toward reduced dementia risk, with rabies (HR=0.43), tetanus & diphtheria & pertussis (Tdap) (HR=0.69), herpes zoster (HR=0.69), influenza (HR=0.74), hepatitis A (HR=0.78), typhoid (HR=0.80), and hepatitis B (HR=0.82) vaccinations being significant. Individuals with more full vaccination types and more annual influenza vaccinations were less likely to develop dementia. Gender and age had no effect on this association.

**Conclusion:**

Routine adult vaccinations are associated with a significant reduction in dementia risk and may be an effective strategy for dementia prevention. Further research is needed to elucidate the causal effects of this association and the underlying mechanisms.

## Introduction

Dementia is a syndrome that manifests as progressive cognitive decline and is the seventh leading cause of death among all diseases (1). Age is the greatest known risk factor, and the number of people living with dementia is increasing as the global population ages. More than 55 million people worldwide currently suffer from dementia, and its prevalence almost doubles every two decades, with the total number of people with dementia expected to reach 139 million in 2050 (1). Dementia not only adversely affects the physical and psychological well-being, ability to perform daily living activities, and quality of life of the individual, but also has long-lasting negative effects on their caregivers and families. The enormous burden makes dementia one of the greatest challenges to global health and social care in the 21st century (1, 2).

Unfortunately, to date, with the exception of aducanumab, which was approved by the FDA in 2021 for the treatment of Alzheimer’s disease even if its efficacy is highly debated (3), no therapies have emerged that can reverse or cure dementia, so investigating and identifying measures that may reduce the risk of its onset is critical to managing the disease and has a significant impact on individuals and society. The onset of dementia is often caused by the interaction of lifestyle, environmental factors, and genes; 12 modifiable risk/protective factors have been confirmed, including hypertension, diabetes, hearing impairment, excessive alcohol consumption, smoking, obesity, depression, head injury, air pollution, infrequent social contact, lack of physical activity, and low level of education. Modifying these 12 factors may prevent or delay dementia by 40% (4). Moreover, many other modifiable protective/risk factors for dementia have been identified, such as sleep disturbances, sleep duration, Mediterranean diet, marital status, vitamin D deficiency, and cardiovascular disease (5–10). A growing body of research suggests that the immune system and infections play an important role in the development of dementia; bacterial, fungal, and viral infections may cause neurotoxic inflammation and oxidative stress in the brain, which can lead to neurodegeneration (11–13). In addition, many sources of inflammation, such as periodontal disease, may be involved in the development of dementia (14–16). Thus, vaccination, the most effective and affordable method to prevent and control infectious diseases, may have a positive effect on dementia risk.

For years, public health authorities have wanted to dispel the myth that vaccinated people are more likely to develop Alzheimer’s disease. This is because vaccination stimulates the immune system and thus causes inflammation, which is part of the pathology of Alzheimer’s disease. But a more important reason is that the occasional neuropsychiatric disorders occurring after vaccination are over-reported in the media as a cause-and-effect relationship to appeal to public sentiment (17). A previous meta-analysis has found a significantly lower risk of dementia after influenza vaccination, but this study did not further explore the factors that might influence this association (18). In addition, several studies have examined the association between various other common vaccines, such as shingles, diphtheria, and tetanus vaccines, and the risk of dementia (19–24). Nevertheless, the current evidence on the association between vaccination and dementia risk is inconsistent and there is a lack of studies that have comprehensively explored this topic, so we conducted this meta-analysis and systematic review to investigate the effect of different vaccinations on dementia risk, taking into account the influence of age, sex, and dose.

## Materials and Methods

The present systematic review and meta-analysis was conducted according to Preferred Reporting Items for Systematic Reviews and Meta-Analyses (PRISMA 2020) guidelines (25), and the protocol for this study is not registered.

### Search Strategy

Two authors extensively searched the PubMed, Embase, Cochrane Library, and Web of Science for relevant studies published from database inception to November 30, 2021. Subject terms (vaccine(s), vaccination, dementia, Alzheimer disease) and corresponding synonyms were combined to form the search strategy. No filters or language restrictions were used. The specific search strategy for each database is provided in the **Appendix**. We also manually searched reference lists of included studies and relevant reviews to avoid missing additional eligible studies.

### Inclusion and Exclusion Criteria

Studies were considered for inclusion if all the following conditions were met: (a) the study subjects were humans. (b) For cohort studies, there was a vaccinated cohort and a non-vaccinated cohort; for case-control studies, the case group was patients with dementia and the control group was not. Dementia was clearly defined, and the use of diagnosis codes from the patient medical records and standard clinical criteria were acceptable. (c) Studies compared the risk of dementia in vaccinated population versus unvaccinated populations. (d) The study design was a longitudinal study, including cohort or case-control studies.

Studies that met any of the following criteria would be excluded: (a) Literature that did not generate raw data such as reviews, meta-analyses, comments. (b) Non-human studies such as animal models and cells. (c) The intervention is a therapeutic vaccine specifically for dementia or Alzheimer’s disease. (d) Case reports, case series, or research which did not establish a temporal relationship between vaccinations and dementia onset, such as cross-sectional study.

### Study Selection and Data Extract

Two authors independently browsed the titles and abstracts of the retrieved records. Full-text reading was performed for potentially eligible papers. Excluded papers would be checked again by a third author.

For eligible studies, two authors independently extracted the following information: first author, year of publication, sample size, gender, age, region, population source, study period, type of vaccine, measures of identifying dementia and vaccination, study design, effect size, adjusted confounding factors, and duration of follow-up. Any disagreements that arose during the above process were resolved by consensus among all authors.

### Study Quality Assessment

The quality of included studies was assessed using the Newcastle-Ottawa Scale (NOS) (26). For cohort studies, scores were given in terms of selection, comparability, and outcome, while case-control studies were scored in terms of selection, comparability, and exposure, both with a maximum score of nine. The study with scores greater than six was considered high quality studies, otherwise it was considered to be at high risk of bias.

### Statistical Analysis

We calculated the pooled hazard ratio (HR) and the corresponding 95% CI for the dementia in the vaccinated population compared to the non-vaccinated population (27). When both adjusted and crude effect sizes were present, the adjusted effect size was used. Heterogeneity among included studies was assessed by Cochran’s *Q* test and Higgins’ *I^2^* statistics (28). Heterogeneity was considered significant when *I^2^* > 50% or *P*_heterogeneity_ < 0.05, and results of the random-effects model were reported; otherwise, heterogeneity was considered acceptable, and results from the fixed-effects model were reported. Stratified analyses were performed according to vaccine type, dose, gender, age, and type of dementia. Sensitivity analyses were performed by excluding one study at a time to observe changes in the pooled results. In addition, we compared the results of the fixed-effects and random-effects models to check the stability of the pooled results. Egger’s and Begg’s tests were used to quantitatively assess publication bias, and we also observed the symmetry of funnel plots by visual inspection (29, 30). No publication bias was considered to exist when both Egger’s and Begg’s *P*-values were greater than 0.05 and the funnel plot was generally symmetrical; otherwise, we assessed the impact of potentially unpublished studies on the results by the trim-and-fill method (31).

All data synthesis and analysis in this study was done using STATA MP/16.0 (Stata Corp LLC, TX, USA). All P values were two-tailed and less than 0.05 was considered significant.

## Results

**Figure 1:**
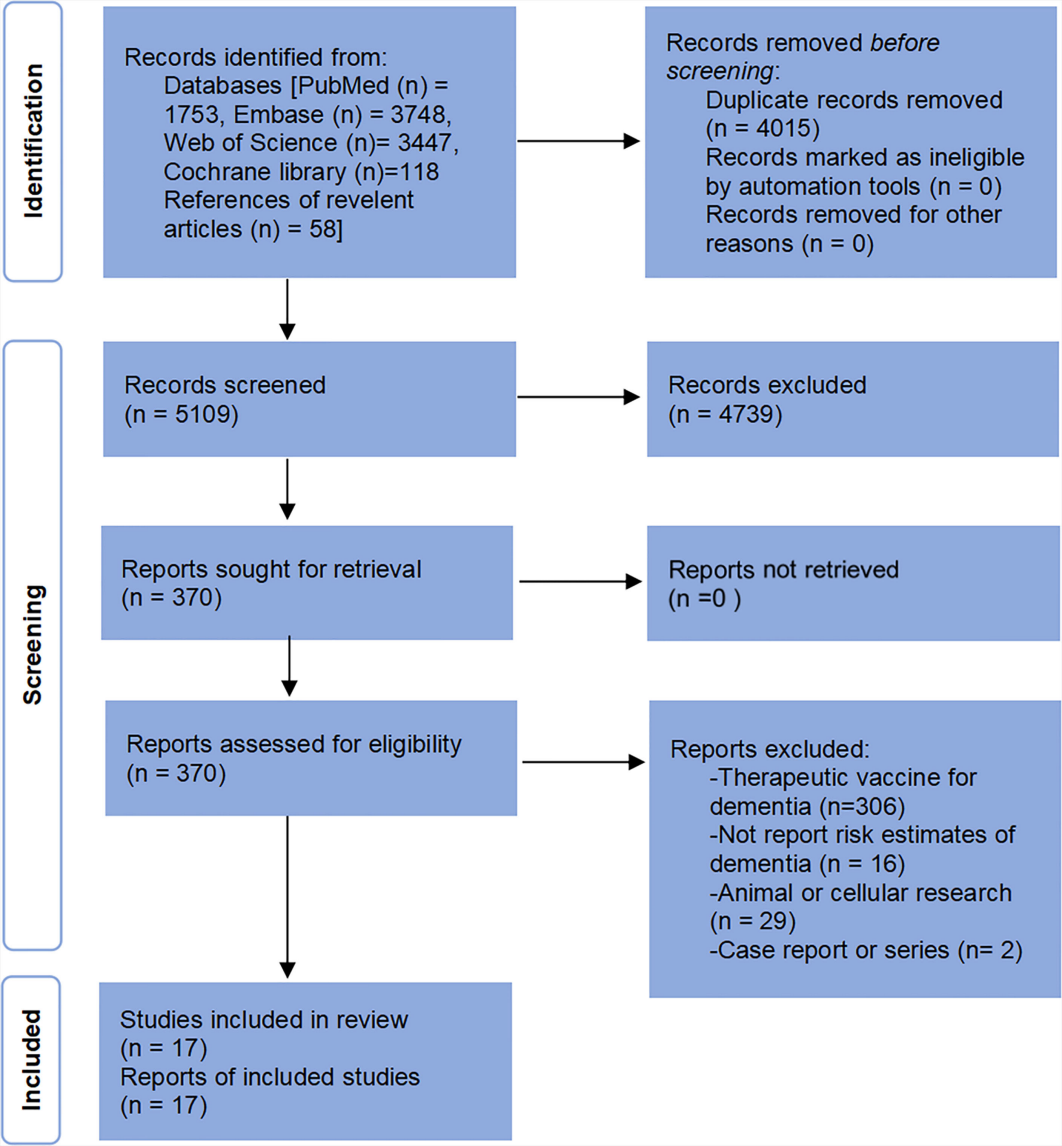
Flow diagram of the study select process.

The pre-developed search strategies yielded a total of 9124 potentially relevant papers. After removing 4015 duplicates, the titles and abstracts of the remaining records were screened, and 4739 clearly non-relevant papers were excluded. Full-text reading was performed on 370 papers, and 17 studies were finally included in this systematic review. The study selection process and the reasons for exclusion after full-text reading are presented in **Figure 1**.

### Description of the Included Studies

A total of 17 studies involving 1857,134 participants assessed the association between common vaccinations and subsequent risk of dementia (19–24, 32–43). All included studies were population-based studies with sample sizes ranging from 1290 to 551344. Eight cohorts were from the United States, four from China, and two each from the United Kingdom, Canada, and Israel. Vaccine types evaluated in more than one cohort for association with dementia risk included influenza, herpes zoster, tetanus & diphtheria & pertussis (Tdap), Bacillus Calmette-Guerin (BCG), pneumonia, and poliomyelitis vaccines. Hepatitis A, hepatitis B, typhoid, rabies, yellow fever, cholera, and meningitis vaccines were also evaluated. 13 studies were retrospective cohort studies, one was a prospective cohort study, and three were nested case-control studies. The median/mean follow-up ranged from 2.9 to 10.6 years, with a maximum follow-up of 20 years. The incidence of dementia in almost all studies was identified according to diagnostic codes, while vaccination was identified according to readable codes and drug names. The detailed characteristics of the included studies are presented in **Table 1**.

**Table 1:** Main characteristics of the included studies.

Author, yr.	Sample (M/F)	Vaccination/case group	Non-vaccination/control group	Mean/median age-yr.	Region	Vaccine type	Population	Study period	Dementia identification	Method of identifying vaccinations	Study design	Confounder adjustment	Mean/median follow-up-yr.
Wiemken (flu) et al., 2021 (32)	123747(118998/4749)	66,822	56,925	75.5	USA	Influenza vaccination	Participants aged ≥65 from VHA	2011- 2019	ICD	CPT, HCPCS codes and drug names	Retrospective cohort study	Age, gender, race, marital status, non-VHA health insurance, geographic region, volume of health care utilization, type 2 diabetes, obesity, hypertension, stroke, ischemic heart disease, congestive heart failure, atrial fibrillation, asthma, chronic obstructive pulmonary disease, traumatic brain injury and vitamin B12 deficiency, depression, anxiety disorder, nicotine dependence and alcohol or drug abuse/dependence, anticholinergics, non-steroidal anti-inflammatory drugs, statins, steroids, antivirals, metformin and sulfonylurea	6.7
Luo, 2020 et al. (33)	19848 (11481/8367)	10980	8868	71.9	Taiwan, China	Influenza vaccination	COPD patients aged ≥60 from NHIRD	2001-2012	ICD	ICD and vaccine drug codes	Retrospective cohort study	Age, sex, urbanization level, monthly income, COPD-related hospitalization, hypertension, dyslipidemia, cerebrovascular diseases, parkinsonism, epilepsy, substance use and alcohol disorder, mood disorder, anxiety disorder, psychotic disorder, sleep disorder	7.4
Liu (CKD) et al.,2016 (34)	11943 (6781/5162)	5745	6198	73.0	Taiwan, China	Influenza vaccination	CKD patients aged ≥60 from NHIRD	2000-2007	ICD	ICD and drug codes	Retrospective cohort study	Age, sex, level of urbanization, and monthly income, diabetes, hypertension, dyslipidemia, cerebrovascular diseases, parkinsonism, epilepsy, substance- and alcohol-use disorders, mood disorder, anxiety disorder, psychotic disorder, sleep disorder	4.4
Lee et al., 2020 (35)	56018 (27117/28901)	22367	33651	<60	Taiwan, China	Influenza vaccination	Periodontitis patients aged ≥50 from NHIRD	2000-2013	ICD	NP	Retrospective cohort study	Age, sex, income, hypertension, mental disorders, diabetes, ischemic heart disease, stroke, hyperlipidemia, COPD, heart failure, liver cirrhosis, traumatic brain injury, renal dialysis, statins, metformin, and follow-up	7.5
Liu (CHF) et al., 2016 (36)	20509	10797	9712	>60	Taiwan, China	Influenza vaccination	CHF patients aged >60 from NHIRD	2001-2012	NP	NP	Retrospective cohort study	NP	NP
Amran et al., 2020 (37)	9066	NP	NP	≥60	USA	Influenza vaccination	Participants aged ≥60 from Cerner Health Fact EHR dataset	NP	ICD	NP	Retrospective cohort study	NP	NP
Tyas et al., 2001 (19)	694(261/433)	36	658	74.0	Canada	Influenza, tetanus, polio, or diphtheria vaccination	Manitoba Study of Health and Aging	1991-1997	NINCDS-ADRDA criteria	Self- reported questionnaire	Nested case–control study	Age, sex, and education	5
Wilkinson et al., 2021 (20)	551344 (275101/276243)	16998	534346	60	UK	Influenza, diphtheria, tetanus, polio, HZ, pneumococcal, rabies, hepatitis A, hepatitis B, hepatitis A+B, hepatitis A +typhoid vaccine, yellow fever, cholera, or meningitis, typhoid vaccination	Participants at age 60 from SAIL Dementia e-cohort	1970-2018	ICD, Read V2-coded primary care data	Read V2-coded primary care data	Nested case–control study	Sex, socioeconomic status, and smoking status	10.6
Verreault et al., 2001 (21)	3865 (1535/2330)	183	3682	≥65	Canada	Influenza, diphtheria, tetanus, or polio vaccination	A representative community sample of elderly Canadians	1991-1997	Diagnosis by physician and the neuropsychologist	Self-reported questionnaire	Prospective cohort study	Age, sex, education, smoking, alcohol consumption, family history of dementia, activities of daily living and instrumental activities of daily living, antecedents of chronic diseases, and perceived health status	5
Scherrer (VHA) et al., 2021 (22)	122946 (118086/4860)	9608	113338	75.6	USA	Tdap vaccination	Participants aged ≥65 from VHA	2010-2019	ICD	CPT codes and product names	Retrospective cohort study	Age, gender, race, and marital status, non-VHA health insurance, type 2 diabetes, obesity, hypertension, stroke, ischemic heart disease, congestive heart failure, atrial fibrillation, asthma, COPD, traumatic brain injury, vitamin B12 deficiency, depression, anxiety disorder, nicotine dependence, and alcohol or drug abuse/dependence, anticholinergics, NSAIDs, statins, steroids, antivirals, metformin, and sulfonylurea, number of well visits, and the volume of total health care utilization	7.9
Scherrer (MarketScan) et al., 2021 (22)	174053(60146/113907)	17872	156181	69.8	USA	Tdap vaccination	Participants aged ≥65 from MarketScan Commercial Claims and Medicare Supplemental databases	2011-2018	ICD	CPT codes and product names	Retrospective cohort study	Age, gender, type 2 diabetes, obesity, hypertension, stroke, ischemic heart disease, congestive heart failure, atrial fibrillation, asthma, COPD, traumatic brain injury, vitamin B12 deficiency, depression, anxiety disorder, nicotine dependence, and alcohol or drug abuse/dependence, anticholinergics, NSAIDs, statins, steroids, antivirals, metformin, and sulfonylurea, number of well visits, and the volume of total health care utilization	3
Wiemken (VHA) et al., 2021 (23)	80070 (76530/3540)	1741	63021	76.8	USA	Both of Tdap and HZ vaccination	Participants aged ≥65 from VHA	2011-2019	ICD	CPT codes and product names	Retrospective cohort study	Age, sex, race, marital status, insurance status, geographic region, overall health care utilization, use of well visits, cancer, type 2 diabetes, obesity, hypertension, stroke, ischemic heart disease, congestive heart failure, atrial fibrillation, asthma, chronic obstructive pulmonary disease, traumatic brain injury, vitamin B12 deficiency, depression, any anxiety disorder, nicotine dependence, alcohol and drug abuse/dependence, antidepressants, benzodiazepines, anticholinergics, NSAIDS, antihypertensives, statins, steroids, antivirals, metformin, sulfonylurea, HZ and Tdap infection	7.1
Wiemken (MarketScan) et al., 2021 (23)	129,200 (44720/84480/)	3791	101,819	70.5	USA	Both of Tdap and HZ vaccination	Participants aged ≥65 from MarketScan Commercial Claims and Medicare Supplemental databases	2012-2018	ICD	CPT codes and product names	Retrospective cohort study	Age, sex, insurance status, geographic region, overall health care utilization, use of well visits, cancer, type 2 diabetes, obesity, hypertension, stroke, ischemic heart disease, congestive heart failure, atrial fibrillation, asthma, chronic obstructive pulmonary disease, traumatic brain injury, vitamin B12 deficiency, depression, any anxiety disorder, nicotine dependence, alcohol and drug abuse/dependence, antidepressants, benzodiazepines, anticholinergics, NSAIDS, antihypertensives, statins, steroids, antivirals, metformin, sulfonylurea, HZ and Tdap infection	2.9
Scherrer (VHA) et al., 2021 (24)	136016 (130550/5466)	27,419	108,597	75.7	USA	HZ vaccination	Participants aged≥65 from VHA	2010-2019	ICD	CPT codes and product names	Retrospective cohort study	Age, sex, race, marital status, non-VHA health insurance, geographic regions, type 2 diabetes, obesity, hypertension, stroke, ischemic heart disease, congestive heart failure, atrial fibrillation, asthma, chronic obstructive pulmonary disease, traumatic brain injury, Vitamin B12 deficiency, depression, anxiety disorder, nicotine dependence, alcohol and drug abuse/dependence, BMI, smoking, anticholinergics, NSAIDS, anti-hypertensives, statins, steroids, antiviral medications, metformin, sulfonylurea, average number of outpatient healthcare encounters/medical claims, HZ infection, and HZ antiviral	7.9
Scherrer (MarketScan) et al., 2021 (24)	172790 (60503/112287)	24612	148178	69.9	USA	HZ vaccination	Participants aged≥65 MarketScan Commercial Claims and Medicare Supplemental databases	2011-2018	ICD	CPT codes and product	Retrospective cohort study	Age, sex, geographic regions, type 2 diabetes, obesity, hypertension, stroke, ischemic heart disease, congestive heart failure, atrial fibrillation, asthma, chronic obstructive pulmonary disease, traumatic brain injury, Vitamin B12 deficiency, depression, anxiety disorder, nicotine dependence, alcohol and drug abuse/dependence, BMI, smoking, anticholinergics, NSAIDS, anti-hypertensives, statins, steroids, antiviral medications, metformin, sulfonylurea, average number of outpatient healthcare encounters/medical claims, HZ infection, and HZ antiviral	3.1
Lophatananon et al., 2021 (38)	228223 (103351/124872)	2378	225845	65.4	UK	HZ vaccination	UK Biobank	2013-2017	ICD and primary care linkage records	Primary care linkage records Read codes	Nested case-control study	Age, sex, and Charlson Comorbidity Index, shingles	≥3
Klinger (CHS) et al., 2021 (39)	6725 (5558/1167)	1578	5147	73.7	Israel	Bacillus Calmette-Guerin	BC patients aged >60 from CHS	2000-2020	ICD	NP	Retrospective cohort study	Age and sex	6.6
Klinger (UCLAH) et al., 2021 (39)	2270 (1680/590)	132	2138	70.3	USA	Bacillus Calmette-Guerin	BC patients aged >60 from UCLAH	Since 2013	ICD	NP	Retrospective cohort study	Age and sex	3.5
Kim et al., 2021 (40)	1290 (910/380)	971	319	71.5	USA	Bacillus Calmette-Guerin	Patients with NMIBC from MHS	1984-2020	ICD	LGCA, MMCCR, and individual chart review	Retrospective cohort study	Age, sex, race, heart disease, cerebrovascular disease, and diabetes	3
Gofrit et al., 2019 (41)	1371 (1134/237)	878	493	68.1	Israel	Bacillus Calmette-Guerin	BC Patients underwent transurethral resection	1966-2018	ICD	NP	Retrospective cohort study	Gender and cancer grade	8
Ukraintseva et al., 2020 (42, 43)	5146	NP	NP	65-75	USA	Pneumococcal with and without an accompanying influenza vaccination	Participants aged 65-75 from Cardiovascular Health Study	NP	NP	NP	Retrospective cohort study	Sex, race, birth cohort, education, smoking, and rs2075650	NP

F/M, female/male; ICD, International Classification of Diseases; CPT, current procedural terminology; HCPCS, Healthcare Common Procedure Coding System; CKD, chronic kidney disease; COPD, chronic obstructive pulmonary disease; CHF, chronic heart failure; NP, not provided; VHA, Veterans Health Administration; NHIRD, Taiwan National Health Insurance Research Database; NINCDS-ADRDA, National Institute of Neurological and Communicative Disorders and Stroke-Alzheimer’s Disease and Related Disorders Association; SAIL, Secure Anonymised Information Linkage databank; CHS, Clalit Health Services; HUH, Hadassah University Hospitals; UCLAH, UCLA Health System; Tdap, tetanus, diphtheria, pertussis; NSAIDs, nonsteroidal anti-inflammatory drugs; HZ, herpes zoster; BC, bladder cancer; MHS, Montefiore Health System; NMIBC, non–muscle-invasive bladder cancer; LGCA, Looking Glass Clinical Analytics; MMCCR, Montefiore Medical Center Cancer Registry.

The overall level of quality of the included studies was excellent, with NOS scores ranging from 7-9 (**Table 2**). Most studies considered the effects of sociodemographics, comorbidities, medications, detection bias, and competing events on dementia risk (**Table 1**).

**Table 2:** The quality assessment of included studies.

Study (cohort)	Representativeness of exposed cohort	Selection of non-exposed cohort	Ascertainment of exposure	Outcome not present before study	Comparability	Assessment of outcome	Follow-up long enough	Adequacy of follow up *	Quality score
Wiemken (Flu) et al., (32)	★	★	★	★	★★	★	★	★	9
Luo et al., (33)	★	★	★	★	★★	★	★	★	9
Liu (CKD), (34)	★	★	★	★	★★	★	★	★	9
Lee et al., (35)	★	★	★	★	★★	★	★	★	9
Liu (CHF) et al., (36)	★	★	★	★	★☆	★	★	☆	7
Amran et al., (37)	★	★	★	★	★☆	★	★	☆	7
Verreault et al., (21)	★	★	★	★	★★	★	★	★	9
Scherrer et al., (22)	★	★	★	★	★★	★	★	★	9
Wiemken (Tdap and HZ), (23)	★	★	★	★	★★	★	★	★	9
Scherrer et al., (24)	★	★	★	★	★★	★	★	★	9
Klinger et al., (39)	★	★	★	★	★★	★	★	★	9
Kim et al., (40)	★	★	★	★	★★	★	★	★	9
Gofrit et al., (41)	★	★	★	★	★★	★	★	★	9
Ukraintseva et al., (43)	★	★	★	★	★★	★	★	☆	8
**Study (case-control)**	**Case definition**	**Representativeness of the cases**	**Selection of Controls**	**Definition of Controls**	**Comparability**	**Ascertainment of exposure**	**Same method of ascertainment**	**Non-Response rate**	**Quality score**
Tyas et al., (19)	★	★	★	★	★★	★	★	★	9
Wilkinson et al., (20)	★	★	★	★	★★	★	★	★	9
Lophatananon et al., (38)	★	★	★	★	★★	★	★	★	9

* Median follow-up of more than 3 years or maximum follow-up of more than 5 years was considered enough.

### Overall Association Between Vaccination and Dementia Risk

**Figure 2:**
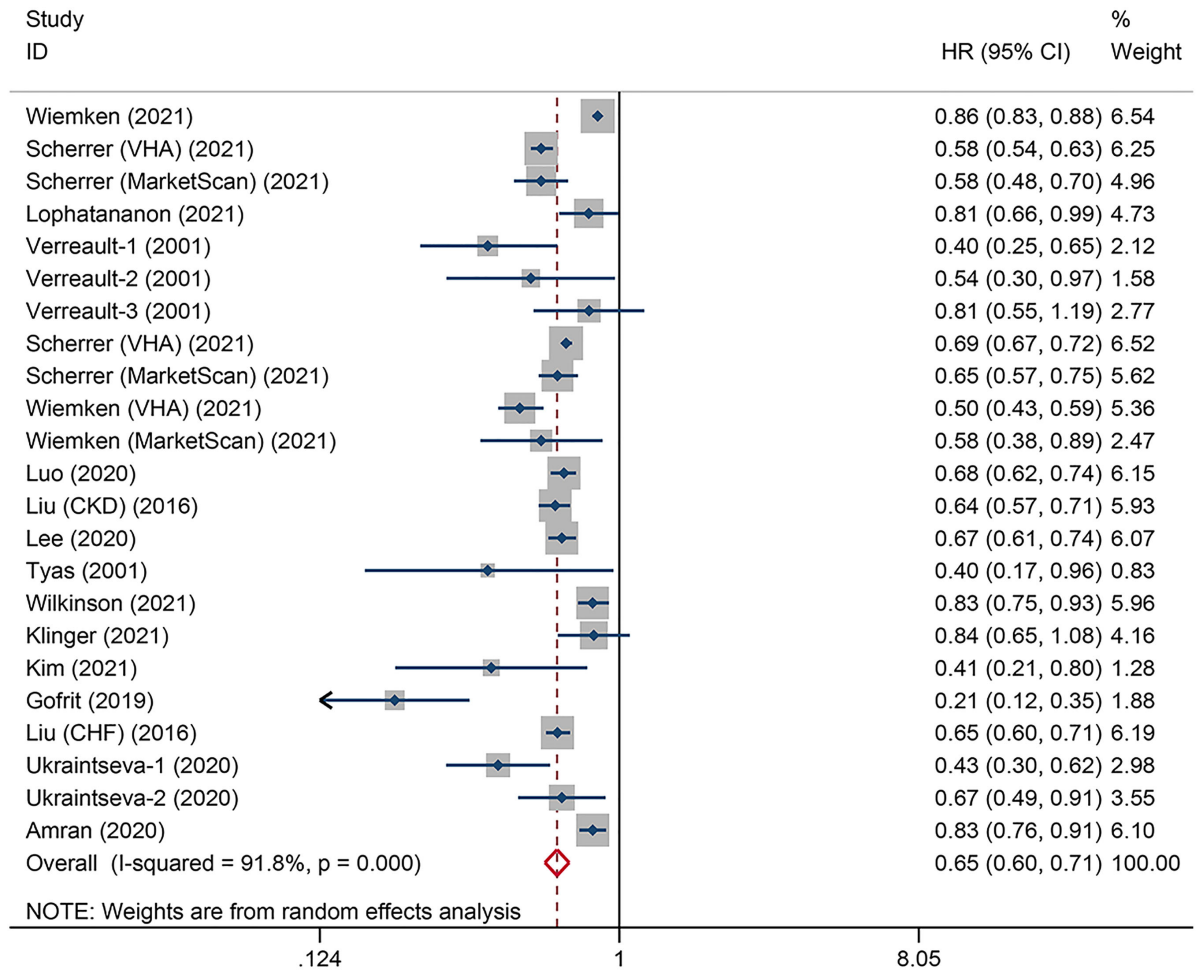
Overall association between adult vaccinations and subsequent dementia risk.

The heterogeneity test showed substantial heterogeneity among individual studies (*I^2 =^* 91.8%, *P*_heterogeneity_<0.001), so the random-effects model was used. The pooled 17 studies was 0.65 (95% CI: 0.60-0.71, *P*_overall effect_ < 0.001), suggesting that vaccinations overall reduced the risk of future dementia by 35% (**Figure 2**).

### Subgroup Analyses

We performed stratified analyses according to vaccine type, gender, age, dose, and type of dementia. Subgroup analyses showed that influenza (HR=0.74, 95% CI: 0.63-0.87, *P*<0.001), herpes zoster (HR=0.69, 95% CI: 0.67-0.72, *P*<0.001), and Tdap (HR=0.69, 95% CI: 0.58-0.82, *P*<0.001) vaccinations and subsequent lower dementia risk were significantly associated. The greatest reduction in dementia risk was seen after BCG vaccination, but statistical significance was borderline (HR=0.42, 95% CI: 0.17-1.07, *P*=0.069). Pneumonia (HR=0.68, 95% CI: 0.41-1.13) and poliomyelitis (HR=0.78, 95% CI: 0.44-1.40) vaccinations also tended to be associated with a lower risk of dementia, but without statistical significance (*P*=0.137 and 0.406, respectively). Furthermore, Wilkinson et al. explored the association between seven other vaccinations and dementia risk using the Secure Anonymised Information Linkage databank containing the population of Wales, UK. All vaccinations were associated with a reduced risk of dementia, with reduced risk ranging from 18% to 57% among those vaccinated against hepatitis A, hepatitis B, typhoid, and rabies, whereas statistical significance was absent for yellow fever, cholera, and meningitis vaccines. Only studies on influenza vaccine explored the effects of vaccination dose on dementia risk. Individuals who received 1 or 2-3 influenza vaccines during the follow-up period had no reduced risk of dementia compared to unvaccinated individuals, whereas people who received at least 4 annual influenza vaccinations had a 49% reduced risk of dementia (HR=0.51, 95% CI: 0.32-0.80, *P*=0.003) (**Table 3**). Moreover, Wiemken et al. found that people who received both Tdap and herpes zoster vaccines had a lower risk of dementia compared to those who received Tdap only or herpes zoster only vaccines (23); Wilkinson et al. found a greater reduced risk of dementia in individuals who received typhoid + hepatitis A vaccinations and hepatitis A + B vaccinations than in those who received only one of these vaccines (20). These findings are suggestive of a dose-response effect. Pooled results did not differ significantly by age, sex, or type of dementia (**Table 3**).

**Table 3:** Subgroup analysis of the association between vaccination and dementia risk.

Subgroups	Studies	HR (95%CI)	*P*_overall effect_	Heterogeneity (*I* ^2^, *P_H_*)	Effects model
**Total**	17	0.65 (0.60, 0.71)	<0.001	91.8%, <0.001	Random
***Vaccine type***					
Influenza	9	0.74 (0.63, 0.87)	<0.001	97.7%, <0.001	Random
Herpes zoster	3	0.69 (0.67, 0.72)	<0.001	10.8%, 0.339	Fixed
Tdap^†^	3	0.69 (0.58, 0.82)	<0.001	97.1%, <0.001	Random
Bacillus Calmette–Guerin	3	0.42 (0.17, 1.07)	0.069	91.5%, <0.001	Random
Pneumonia	2	0.68 (0.41, 1.13)	0.137	92.8%, <0.001	Random
Poliomyelitis	2	0.78 (0.44, 1.40)	0.406	73.6%, 0.052	Random
Other	1	0.78 (0.74, 0.81)	<0.001	21.0%, 0.256	Fixed
***Gender***					
Male	5	0.66 (0.58, 0.74)	<0.001	56.8%, 0.055	Random
Female	5	0.67 (0.63, 0.72)	<0.001	0.0%, 0.911	Fixed
***Age***					
<70 years	5	0.74 (0.66, 0.84)	<0.001	72.6%, 0.001	Random
≥70 years	7	0.64 (0.57, 0.72)	<0.001	94.6%, <0.001	Random
***Dose* ^‡^**					
1 vaccine	3	1.03 (0.98, 1.08)	0.229	6.9%, 0.342	Fixed
2-3 vaccine	3	0.87 (0.74, 1.02)	0.088	88.9%, <0.001	Random
≥4 vaccine	4	0.51 (0.32, 0.80)	0.003	98.7%, <0.001	Random
***Dementia type***					
Alzheimer’s disease	10	0.63 (0.55, 0.72)	<0.001	87.9%, <0.001	Random
Vascular dementia	1	0.60 (0.45, 0.80)	<0.001	NA	NA
Other dementia	1	0.69 (0.62, 0.75)	<0.001	NA	NA

HR, hazard risk; OS, overall survival; DFS, disease-free survival; NA, not applicable; Tdap: tetanus, diphtheria, pertussis.

^†^This analysis group included cohorts that reported receiving one or two of the Tdap vaccines.

**^‡^** Only the studies on influenza vaccines supported dose grouping.

### Evaluation for Publication Bias

**Figure 3:**
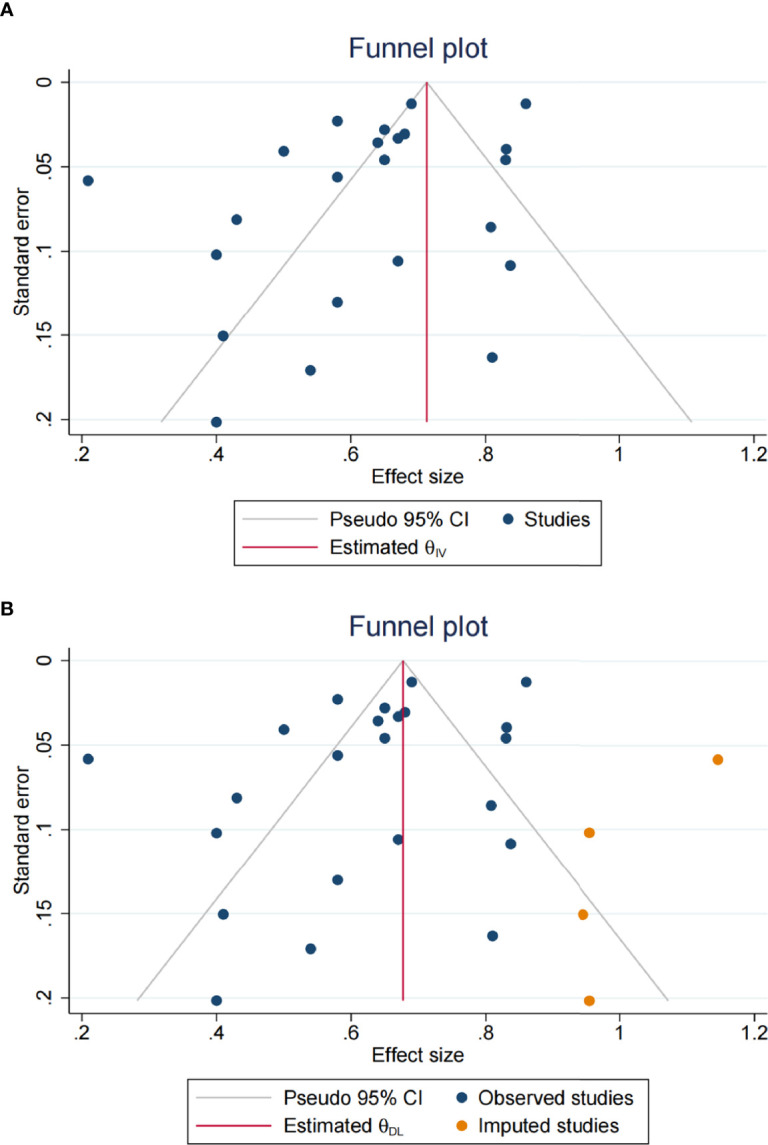
Publication bias assessment. **(A)** The funnel plot; **(B)** four hypothetical studies were observed through the trim-and-fill method.

The *P*-values of Begg’s and Egger’s tests were 0.6343 and 0.2127, respectively, indicating a low possibility of publication bias. However, the funnel plot exhibited a slight asymmetry, so publication bias was still suspected (**Figure 3A**). The trim-and-fill analysis estimated that four hypothetical studies were missing (**Figure 3B**). When four studies were imputed and added to the meta-analysis, the overall HR was 0.677 (95% CI: 0.598-0.755, *P*<0.001), indicating that the effect of potentially unpublished studies on the current pooled results was subtle.

### Sensitivity Analysis

**Figure 4:**
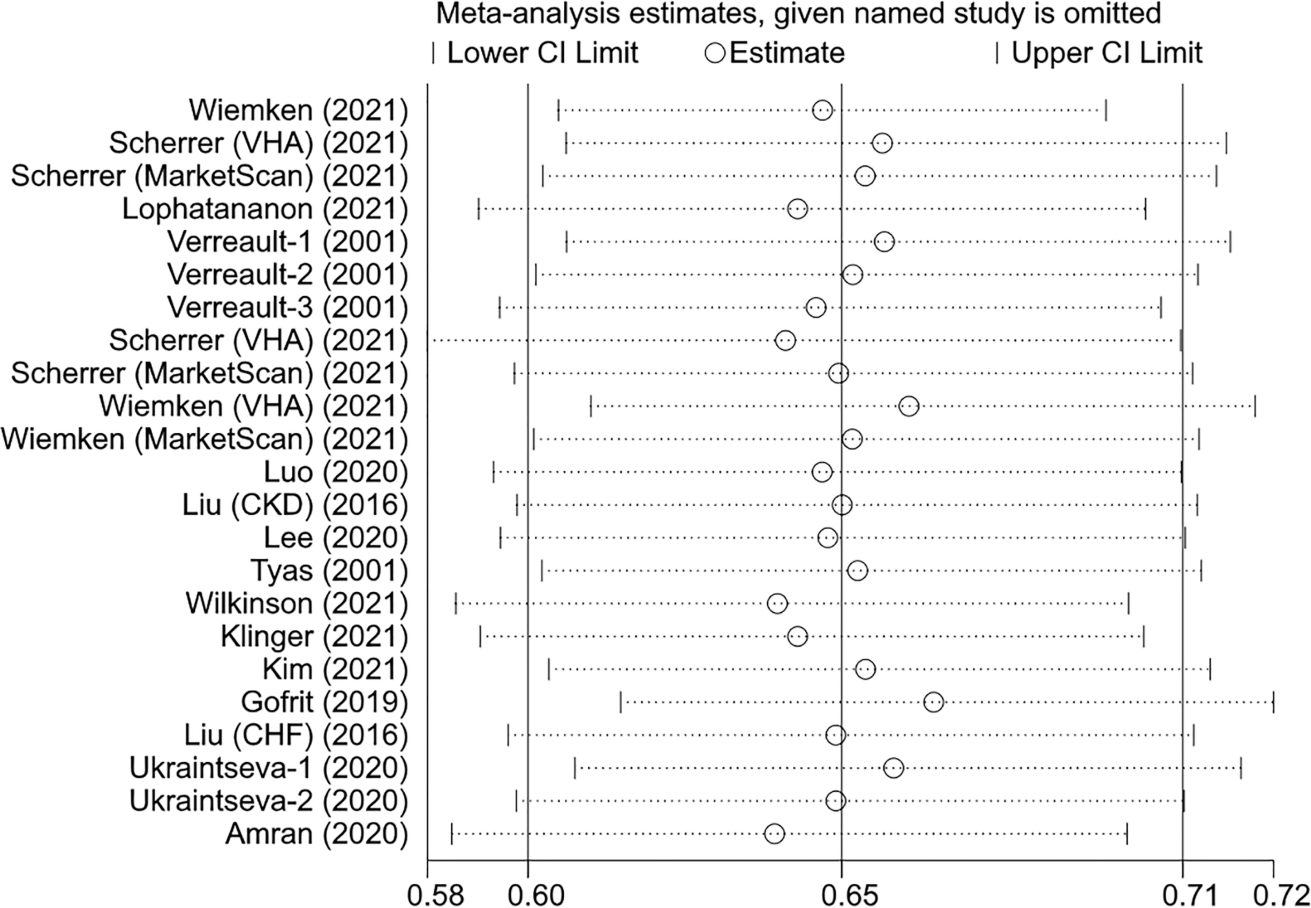
The effects of the individual studies on the overall pooled result.

We assessed the effect of individual studies on the overall result through excluding one study at a time and then combining the remaining studies. The pooled HR did not change significantly when any single study was excluded, indicating that the current overall result is robust (**Figure 4**). Furthermore, we examined the robustness of each meta-analysis by comparing the results of random- and fixed-effects models. The conclusions remained unchanged in all groups except for the 2-3 dose and BCG groups where their results shifted from non-significant to significant (**Table 4**).

**Table 4:** Comparison of the results of random-effects vs. fixed-effects models.

Analysis groups	HR (95% CI), random-effects model	HR (95% CI), fixed-effects model
Total	0.65 (0.60, 0.71)	0.74 (0.73, 0.76)
Influenza vaccination	0.74 (0.63, 0.87)	0.89 (0.87, 0.91)
Herpes zoster vaccination	0.69 (0.66, 0.73)	0.69 (0.67, 0.72)
Tdap vaccination	0.69 (0.58, 0.82)	0.80 (0.77, 0.84)
BCG vaccination	0.42 (0.17, 1.07)	0.62 (0.50, 0.76)
Pneumonia vaccination	0.68 (0.41, 1.13)	1.00 (0.96, 1.03)
Poliomyelitis vaccination	0.78 (0.44, 1.40)	0.95 (0.80, 1.11)
Other vaccination	0.77 (0.73, 0.81)	0.78 (0.74, 0.81)
Male	0.66 (0.58, 0.74)	0.65 (0.60, 0.69)
Female	0.67 (0.63, 0.72)	0.67 (0.63, 0.72)
<70 years	0.74 (0.66, 0.84)	0.74 (0.70, 0.78)
≥70 years	0.64 (0.57, 0.72)	0.72 (0.71, 0.74)
1 dose vaccine	1.03 (0.98, 1.08)	1.03 (0.98, 1.08)
2-3 dose vaccine	0.87 (0.74, 1.02)	0.94 (0.91, 0.98)
≥4 dose vaccine	0.51 (0.32, 0.80)	0.65 (0.62, 0.68)
Alzheimer’s disease	0.63 (0.55, 0.72)	0.78 (0.75, 0.81)

## Discussion

This systematic review and meta-analysis comprehensively investigated the current evidence regarding the effect of routine adult vaccinations on the risk of dementia, and the pooled results from 17 studies with more than 1.8 million participants showed a 35% reduction in the dementia risk after vaccinations. This reduction was observed to be significant in influenza, herpes zoster, Tdap, hepatitis A, hepatitis B, typhoid, and rabies vaccinations, with similar trends but not significant in other vaccines. The reduced risk did not vary with age, gender, or type of dementia. Compared to the previous meta-analysis on influenza vaccination conducted by Veronese et al, we added four new studies related to influenza vaccination and overall pooled results were similar. Interestingly, we found a dose-response effect of vaccination on the incidence of dementia. More full vaccination types and a greater number of annual influenza vaccinations were associated with a lower risk of dementia.

The exact biological mechanism underlying the association between vaccination and reduced risk of dementia is unclear. One possible explanation is that vaccination can reduce the probability of developing infectious diseases. Studies have shown that infectious events may impair cognition and increase the risk of dementia (44, 45). A large multi-cohort study suggests that infections causing an increased risk of dementia are not limited to central nervous system infections, which suggested that the systemic effects of general inflammation may affect the brain (11). Recent research suggests that even infectious diseases like COVID-19, which is dominated by respiratory symptoms, may still be associated with structural changes in the brain and cognitive decline (46–49). However, the association between vaccinations and dementia risk may not be fully explained by preventing the relevant pathogens, as the efficacy of vaccines against different pathogens is not equal but significant reductions in dementia risk can be observed after almost all types of vaccination. In addition, two studies found no attenuation of the association between vaccinations and reduced risk of dementia after adjusting for infection with vaccine-associated pathogens (23, 24). Hence, another possible theory is the non-specific effects (NSEs), also called off-target effects (50). Vaccinations may enable the body’s immune system to be trained and maintain appropriate immune responses thereby increasing the ability to resist bacteria and viruses from entering the brain and reducing the risk of dementia caused by chronic inflammation and abnormal neuroinflammation. Studies have found that multiple basic vaccinations reduced all-cause mortality in children, which supports the NSEs for vaccines (51). The dose-dependent relationship further supports the NSEs hypothesis. Furthermore, specific vaccines may also affect dementia onset through other unique mechanisms. For example, the influenza vaccine reduces cerebrovascular events, a known risk factor for dementia (52, 53); the herpes zoster vaccine prevents reactivation of herpes virus in the brain; and intravesical BCG instillation significantly increases serum IL-2 levels, which leads to an expansion of the number of neuroprotective Treg cells (54, 55). In fact, previous studies have also shown that influenza and BCG vaccines in animal models can enhance and maintain microglia activation, restore brain immune homeostasis and reduce Aβ burden, ultimately improving cognitive impairment (56, 57). It is therefore possible that these vaccines have preventive benefits by intervening in the pathological process of dementia.

It may also be argued that the apparent protective effect of vaccination simply reflects the different health characteristics and lifestyles between vaccinated population and unvaccinated population, such as better knowledge of disease prevention, higher education and income, and greater social support (58). Of note, most of the included studies adjusted for demographics, comorbidities, and medications use variables. Moreover, several studies considered detection bias and socioeconomic factors such as income, marital status, insurance status, urbanization, and education. Wiemken et al. also explored the effect of health adherence bias, and their findings remained unchanged, with little change in the effect size (23). However, unmeasured or residual confounding may still bias the analyses in the original studies. Dementia is a chronically progressive disease, and mild cognitive decline may exist years before dementia is formally diagnosed. It is also possible that people with reduced cognitive reserve are more likely to neglect vaccination. Wilkinson et al. conducted a stratified analysis based on follow-up time, and interestingly, all vaccine types had lower HRs for dementia ten years after vaccination than five years after vaccination, suggesting that the reverse causality is less likely (20). However, given the lack of prospective randomized controlled studies, the current evidence is not sufficient to conclude any causal effect.

Even if the evidence from observational studies cannot definitively show that vaccinations will prevent dementia, the benefits of timely and full vaccination for adults are still substantial. As the body’s immunity declines with age, especially in people with underlying diseases such as hypertension, coronary heart disease, and diabetes, following the recommended immunization schedule not only prevents vaccine-related infectious diseases, but also prevents the exacerbation of existing chronic diseases due to infections (59). Moreover, a recent nationwide prospective study from Denmark found that patients with dementia had a significantly higher mortality rate following infections compared to people without dementia, more than six times that of people with neither dementia nor infection; this markedly increased risk can persist for up to 10 years (60). Therefore, the full complement of recommended vaccines is critical for both the general elderly and those already suffering from dementia. Vaccinations now recommended by the U.S. Centers for Disease Control and Prevention for people aged≥65 include influenza, Tdap, varicella, zoster, pneumococcal, meningococcal, hepatitis A, hepatitis B, haemophilus influenzae type b, and COVID-19 vaccines (61). Of note, although there are currently no studies on the effect of COVID-19 vaccines on dementia risk, given the multiple neurological complications of SARS-CoV-2, COVID-19 vaccination may be beneficial in preventing cognitive decline, but future studies are needed to confirm this (62). Reports from different regions indicate that coverage of influenza vaccine, the recommended annual vaccine, is low among older adults (63, 64). This suggests the need for specific interventions to increase vaccination coverage in older adults. In addition to making vaccination as a key component of healthy aging, vaccination could also be considered as an important component of dementia prevention strategies, like losing weight, stopping smoking, limiting alcohol, and regular exercise.

Several limitations of this study need to be considered. The number of studies exploring the association between certain vaccine types and dementia risk is small, so it is not clear whether this association varies by region and ethnicity. The populations in some studies were limited to those with specific conditions, such as chronic kidney failure, chronic obstructive pulmonary disease, and bladder cancer. Furthermore, the predominant type of vaccine used against the same pathogens differed across time and regions, for example, there were inactivated, recombinant, and live attenuated influenza vaccines. These may have contributed to the heterogeneity of this meta-analysis. Finally, current findings may be affected by publication bias, but the conclusions remain unchanged after adding hypothetical unpublished studies.

## Conclusion

Our study shows that routine vaccinations are significantly associated with dementia risk reduction and more vaccinations have a stronger protective effect on dementia development. Vaccination may be an inexpensive and accessible intervention to prevent cognitive decline and deserve to be incorporated into the management of dementia prevention strategies. Prospective studies and basic experiments are needed to clarify the causal relationship and underlying mechanisms of this association.

## Data Availability Statement

The original contributions presented in the study are included in the article/**Supplementary Material**. Further inquiries can be directed to the corresponding author.

## Author Contributions

Conception and design: ZS and ML. Database search, study selection, and data extraction: XW and SH, Statistical analysis: TX and DC. Drafting of manuscript: XW, HY, ML, YZ, and JL. Editing and critical revision of the manuscript: ZS and ML. All authors contributed to the article and approved the submitted version.

## Conflict of Interest

The authors declare that the research was conducted in the absence of any commercial or financial relationships that could be construed as a potential conflict of interest.

## Publisher’s Note

All claims expressed in this article are solely those of the authors and do not necessarily represent those of their affiliated organizations, or those of the publisher, the editors and the reviewers. Any product that may be evaluated in this article, or claim that may be made by its manufacturer, is not guaranteed or endorsed by the publisher.
