# Supplementary material for: Adult Vaccination as a Protective Factor for Dementia: A Meta-Analysis and Systematic Review of Population-Based Observational Studies
Source: Front Immunol. 2022 May 3;13:872542. doi: 10.3389/fimmu.2022.872542 (PMC9110786; doi:10.3389/fimmu.2022.872542)
Supplement: Supplementary file 1 [file DataSheet_1.docx]

**PubMed:**

(("Vaccines"[Mesh] or vaccine) OR ("Vaccination"[Mesh] or Vaccinations or Immunization, Active or Active Immunization or Active Immunizations or Immunizations, Active)) AND ((("Dementia"[Mesh]) or Dementias or Amentia or Amentias or Senile Paranoid Dementia or Dementias, Senile Paranoid or Paranoid Dementia, Senile or Paranoid Dementias, Senile or Senile Paranoid Dementias or Familial Dementia or Dementia, Familial or Dementias, Familial or Familial Dementias) OR (("Alzheimer Disease"[Mesh]) or Alzheimer Dementia or Alzheimer Dementias or Dementia, Alzheimer or Alzheimer's Disease or Dementia, Senile or Senile Dementia or Dementia, Alzheimer Type or Alzheimer Type Dementia or Alzheimer-Type Dementia (ATD) or Alzheimer Type Dementia (ATD) or Dementia, Alzheimer-Type (ATD) or Alzheimer Type Senile Dementia or Primary Senile Degenerative Dementia or Dementia, Primary Senile Degenerative or Alzheimer Sclerosis or Sclerosis, Alzheimer or Alzheimer Syndrome or Alzheimer's Diseases or Alzheimer Diseases or Alzheimers Diseases or Senile Dementia, Alzheimer Type or Acute Confusional Senile Dementia or Senile Dementia, Acute Confusional or Dementia, Presenile or Presenile Dementia or Alzheimer Disease, Late Onset or Late Onset Alzheimer Disease or Alzheimer's Disease, Focal Onset or Focal Onset Alzheimer's Disease or Familial Alzheimer Disease (FAD) or Alzheimer Disease, Familial (FAD) or Familial Alzheimer Diseases (FAD) or Alzheimer Disease, Early Onset or Early Onset Alzheimer Disease or Presenile Alzheimer Dementia))

**Embase:**

(('vaccine'/exp or 'combined vaccine' or vaccin or 'vaccine control' or vaccine or vaccines or 'vaccines, combined') OR ('vaccination'/exp or 'vaccination policy' or 'vaccination program' or 'vaccination programme' or 'vaccinotherapy' or 'virus vaccination')) AND (('dementia'/exp or amentia or demention) OR ('Alzheimer disease'/exp or 'Alzeimer disease' or 'Alzeimers disease' or 'Alzheimer dementia' or 'alzheimer fibrillary change' or 'alzheimer fibrillary lesion' or 'alzheimer neurofibrillary change' or 'alzheimer neurofibrillary degeneration' or 'alzheimer neuron degeneration' or 'alzheimer perusini disease' or 'alzheimer sclerosis' or 'alzheimer syndrome' or 'cortical sclerosis, diffuse' or 'dementia, alzheimer' or 'diffuse cortical sclerosis' or 'late onset alzheimer disease'))

**Web of Science:**

TS=(((Dementia or Dementias or Amentia or Amentias or Senile Paranoid Dementia or Dementias, Senile Paranoid or Paranoid Dementia, Senile or Paranoid Dementias, Senile or Senile Paranoid Dementias or Familial Dementia or Dementia, Familial or Dementias, Familial or Familial Dementias or demention) OR (Alzheimer Disease or Alzheimer Dementia or Alzheimer Dementias or Dementia, Alzheimer or Alzheimer's Disease or Dementia, Senile or Senile Dementia or Dementia, Alzheimer Type or Alzheimer Type Dementia or Alzheimer-Type Dementia (ATD) or Alzheimer Type Dementia (ATD) or Dementia, Alzheimer-Type (ATD) or Alzheimer Type Senile Dementia or Primary Senile Degenerative Dementia or Dementia, Primary Senile Degenerative or Alzheimer Sclerosis or Sclerosis, Alzheimer or Alzheimer Syndrome or Alzheimer's Diseases or Alzheimer Diseases or Alzheimers Diseases or Senile Dementia, Alzheimer Type or Acute Confusional Senile Dementia or Senile Dementia, Acute Confusional or Dementia, Presenile or Presenile Dementia or Alzheimer Disease, Late Onset or Late Onset Alzheimer Disease or Alzheimer's Disease, Focal Onset or Focal Onset Alzheimer's Disease or Familial Alzheimer Disease (FAD) or Alzheimer Disease, Familial (FAD) or Familial Alzheimer Diseases (FAD) or Alzheimer Disease, Early Onset or Early Onset Alzheimer Disease or Presenile Alzheimer Dementia)) AND ((vaccine or vaccines or vaccin) OR (vaccination or vaccination policy or vaccination program or vaccination programme or vaccinotherapy or virus vaccination)))

**Cochrane:**

ID        Search

#1        MeSH descriptor: [Vaccines] explode all trees

#2        Vaccine

#3        MeSH descriptor: [Vaccination] explode all trees

#4        Active Immunizations or Immunizations, Active or Immunization, Active or Active Immunization or Vaccinations

#5        MeSH descriptor: [Dementia] explode all trees

#6        Dementias, Familial or Familial Dementia or Familial Dementias or Dementia, Familial or Dementias or Amentia or Amentias or Senile Paranoid Dementia or Paranoid Dementias, Senile or Paranoid Dementia, Senile or Senile Paranoid Dementias or Dementias, Senile Paranoid

#7        MeSH descriptor: [Alzheimer Disease] explode all trees

#8        Alzheimer Sclerosis or Senile Dementia or Alzheimer Type Dementia or Alzheimer Dementias or Dementia, Alzheimer-Type (ATD) or Alzheimer Type Senile Dementia or Dementia, Senile or Dementia, Primary Senile Degenerative or Primary Senile Degenerative Dementia or Alzheimers Diseases or Alzheimer's Diseases or Alzheimer Type Dementia (ATD) or Alzheimer Diseases or Alzheimer's Disease or Dementia, Alzheimer or Sclerosis, Alzheimer or Senile Dementia, Alzheimer Type or Alzheimer Dementia or Alzheimer-Type Dementia (ATD) or Alzheimer Syndrome or Dementia, Alzheimer Type or Alzheimer Disease, Late Onset or Late Onset Alzheimer Disease or Dementia, Presenile or Presenile Dementia or Alzheimer's Disease, Focal Onset or Focal Onset Alzheimer's Disease or Acute Confusional Senile Dementia or Senile Dementia, Acute Confusional or Presenile Alzheimer Dementia or Alzheimer Disease, Early Onset or Early Onset Alzheimer Disease or Alzheimer Disease, Familial (FAD) or Familial Alzheimer Disease (FAD) or Familial Alzheimer Diseases (FAD)

#9        #1 or #2 or #3 or #4

#10      #5 or #6 or #7 or #8

#11      #9 and #10
